# Evaluation of the Influence of Er:YAG Laser Parameters on the Effectiveness of Growth Inhibition of *Candida* Biofilms: An In Vitro Study

**DOI:** 10.3390/jcm15010018

**Published:** 2025-12-19

**Authors:** Diana Dembicka-Mączka, Jakub Fiegler-Rudol, Małgorzata Kępa, Dariusz Skaba, Rafał Wiench

**Affiliations:** 1 Dental Office, Artistic Smile Studio, 61/1 Krakowska Street, 33-100 Tarnów, Poland; 2 Department of Periodontal and Oral Mucosa Diseases, Faculty of Medical Sciences in Zabrze, Medical University of Silesia, 40-055 Katowice, Polandrwiench@sum.edu.pl (R.W.); 3 Department of Microbiology, Faculty of Pharmaceutical Sciences in Sosnowiec, Medical University of Silesia, 41-200 Sosnowiec, Poland; mkepa@sum.edu.pl

**Keywords:** Er:YAG lasers, biofilms, Candida, oral candidiasis, laser therapy, in vitro techniques

## Abstract

**Background/Objectives**: *Candida* biofilms exhibit high resistance to antifungal treatment, motivating investigation of adjunctive physical disinfection methods. To quantitatively assess the effect of Er:YAG laser fluence on growth inhibition and viability of single-species *Candida* biofilms in vitro using a 7 mm full-beam handpiece. **Methods**: Biofilms of *Candida albicans* ATCC 10231, *C. glabrata* ATCC 90030, *C. parapsilosis* ATCC 22019, and *C. krusei* ATCC 6258 were grown on Sabouraud agar. In phase 1, growth inhibition zones (GIZs) were evaluated after non-contact Er:YAG irradiation (2 Hz, 300 µs, 10 mm distance, no air or water spray) at fluences from 0.3 to 3.4 J/cm^2^, with incubation for 24 to 96 h. In phase 2, 96 h mature biofilms were irradiated for 120 s at 0.8, 1.0, 1.5, or 2.0 J/cm^2^, and viability was quantified by colony-forming unit (CFU) imprinting. All experimental conditions were tested in quadruplicate. **Results**: GIZ diameters increased significantly with fluence for all species (*p* < 0.05) and remained stable up to 96 h. At the highest fluence, mean GIZs reached approximately 8.0 mm for *C. albicans*, 7.7 mm for *C. parapsilosis*, 7.0 mm for *C. krusei*, and 5.2 mm for *C. glaxfbrata*. In mature biofilms, CFU counts decreased significantly with increasing fluence (*p* < 0.05). For *C. albicans*, CFUs were reduced from 164.0 ± 25.1 at 0.8 J/cm^2^ to 16.5 ± 5.2 at 2.0 J/cm^2^, while *C. glabrata* decreased from 103.5 ± 5.4 to 20.8 ± 1.7. *C. parapsilosis* and *C. krusei* showed maximal reductions at 1.0–1.5 J/cm^2^, followed by partial CFU rebound at 2.0 J/cm^2^. **Conclusions**: Er:YAG irradiation delivered over a large, uniformly illuminated area induces stable, fluence-dependent inhibition and significant reduction of *Candida* biofilm viability in vitro. Optimal fluence ranges are species specific, underscoring the need for parameter optimization and further evaluation in more complex biofilm models before clinical extrapolation.

## 1. Introduction

### 1.1. Background

Fungal infections caused by yeasts of the genus *Candida* represent a significant clinical problem, particularly in immunocompromised patients, individuals with diabetes, those receiving prolonged antibiotic therapy, and denture wearers [1,2,3]. *Candida albicans*, *C. glabrata*, *C. parapsilosis*, and *C. krusei* are among the species most frequently associated with oral candidiasis and denture-related stomatitis [1,2]. A major virulence factor of these organisms is their ability to form biofilms, structured microbial communities embedded in an extracellular matrix composed of polysaccharides, proteins, and extracellular DNA [4]. Biofilm formation markedly increases tolerance to antifungal agents and environmental stress. *Candida* cells within biofilms have been shown to be up to several hundred-fold more resistant to commonly used antifungals, such as fluconazole and amphotericin B, than their planktonic counterparts [3,4]. Consequently, conventional pharmacological therapy often achieves only partial reduction of fungal burden rather than complete eradication, contributing to persistence and recurrence of infection [2,5]. These limitations have driven interest in adjunctive physical disinfection approaches that may reduce biofilm-associated microorganisms without relying exclusively on antifungal drugs.

### 1.2. Er:YAG Laser and Antimicrobial Applications

The Er:YAG (erbium:yttrium–aluminum–garnet) laser emits radiation at a wavelength of 2940 nm, which is strongly absorbed by water and hydroxyapatite [6,7]. This characteristic underlies its widespread clinical use in dentistry and has also prompted investigation of its antimicrobial potential. Absorption of laser energy by water-rich microbial cells and biofilm matrices can induce rapid heating, micro-expansive effects, and structural disruption, resulting in decreased microbial viability [7,8,9,10]. Several in vitro studies have demonstrated that Er:YAG irradiation can reduce bacterial and fungal biofilms on dental hard tissues, implant surfaces, and restorative materials [11,12,13,14,15]. However, most available data focus on bacterial species, while evidence concerning *Candida* biofilms remains comparatively limited. Studies addressing *Candida* have often used narrow fiber tips, relatively high fluence values, or short exposure times, and have primarily examined early biofilm stages rather than mature biofilms [10,11,12,13]. Importantly, reported outcomes generally indicate partial reductions in viable counts rather than complete elimination, suggesting that Er:YAG irradiation acts as a disinfection modality rather than achieving sterilization [10,15,16].

### 1.3. Rationale and Aim of the Study

Despite increasing interest in laser-based disinfection, there is a lack of systematic data on the response of different *Candida* species, particularly mature biofilms, to Er:YAG irradiation delivered at low fluence over a large, homogeneously illuminated area. Studies specifically investigating the influence of beam diameter and uniform energy distribution on antifungal effectiveness are scarce [10,16,17]. As a result, optimal parameter ranges for reproducible and species-specific biofilm reduction remain insufficiently defined. The aim of this in vitro study was therefore to evaluate the influence of selected Er:YAG laser parameters on growth inhibition and viability reduction of single-species *Candida* biofilms. Particular emphasis was placed on low fluence irradiation delivered with a 7 mm full-beam handpiece and on comparing responses among clinically relevant *Candida* species. The working hypothesis was that Er:YAG irradiation under these conditions would produce a significant, fluence-dependent reduction in biofilm growth and viability compared with non-irradiated controls. The null hypothesis was that laser exposure would not result in statistically significant differences in growth inhibition zones or colony-forming unit counts. While Candida-associated biofilm infections represent an important clinical challenge, the present investigation is designed as a preliminary in vitro study. Its purpose is to generate controlled experimental data on fluence-dependent Er:YAG laser effects that may inform the design of future in vivo and clinical studies, rather than to establish immediate clinical applicability.

## 2. Materials and Methods

### 2.1. Study Design

This in vitro study was designed to evaluate the fluence-dependent effects of Er:YAG laser irradiation on single-species *Candida* biofilms using two complementary experimental endpoints. The study consisted of two sequential experimental phases addressing distinct, but related, outcome measures. Phase 1 (growth inhibition phase) assessed the ability of Er:YAG irradiation to inhibit radial expansion of *Candida* biofilms formed on solid agar. The outcome of this phase was the diameter of the growth inhibition zone (GIZ), measured after laser exposure and subsequent incubation. This phase was intended to characterize fluence-dependent suppression of biofilm growth over time. Phase 2 (biofilm viability reduction phase) evaluated the effect of Er:YAG irradiation on established, mature *Candida* biofilms. The outcome of this phase was the number of viable microorganisms remaining after irradiation, quantified as colony-forming units (CFUs) obtained by contact imprinting. This phase focused on assessing laser-induced disinfection rather than eradication.

The primary outcome variables were:

1. Diameter of the growth inhibition zone (GIZ) in Phase 1
2. CFU counts following irradiation of mature biofilms in Phase 2

The secondary outcomes included:

- Persistence of inhibition zones over a 96 h incubation period
- Species-specific differences in fluence–response relationships

The independent variable in both phases was laser fluence, delivered at a fixed pulse frequency of 2 Hz and pulse duration of 300 µs. The dependent variables were GIZ diameter and CFU count, respectively. Each experimental condition, defined by *Candida* species and laser setting, was tested in four independent replicates (*n* = 4). The sample size was selected based on consistency with prior in vitro laser disinfection studies and preliminary experiments indicating low within-group variability. A formal a priori power calculation was not performed; however, the chosen replication level was sufficient to detect statistically significant differences between fluence levels using one-way analysis of variance at a significance level of *p* < 0.05.

### 2.2. Microorganisms

The study included reference strains obtained from the American Type Culture Collection (ATCC): *Candida albicans* ATCC 10231, *C. glabrata* ATCC 90030, *C. parapsilosis* ATCC 22019, and *C. krusei* ATCC 6258. These species were selected due to their established relevance in oral candidiasis and denture-related infections [1,2]. All strains originated from the culture collection of the Department of Microbiology, Faculty of Pharmaceutical Sciences, Medical University of Silesia (Sosnowiec, Poland), and were stored at −80 °C in tryptic soy broth supplemented with glycerol until use. Only reference strains were used in this study; no clinical isolates were included. While this approach allows standardized inter-species comparisons and reproducibility, it represents a limitation with respect to external validity and strain-level heterogeneity, which is addressed further in Section 4.

### 2.3. Growth Conditions

Prior to experimentation, frozen stocks were thawed and subcultured twice on Sabouraud dextrose agar supplemented with chloramphenicol to ensure viability and purity. Each subculture was incubated for 24 h at 37 °C. After the second subculture, a fresh overnight culture was used to prepare cell suspensions. Colonies were suspended in sterile 0.9 percent NaCl solution and adjusted to a density of 0.5 McFarland standard, corresponding to approximately 1 × 10^6^ cells/mL, as measured at 525 nm using a DensiLa-Meter II device (Brno, Czech). The standardized suspensions were used for inoculation within 15 min of preparation to minimize variability related to cell settling or growth phase changes. For biofilm formation, standardized suspensions were evenly inoculated onto Sabouraud dextrose agar plates and incubated at 37 °C under aerobic conditions. Biofilms intended for the growth inhibition phase were irradiated immediately after inoculation, whereas biofilms intended for the mature biofilm phase were incubated for 96 h prior to laser exposure.

### 2.4. Experimental Groups

For each *Candida* species, Petri dishes containing Sabouraud dextrose agar were assigned to one of the following groups:

- Control group: Biofilms not exposed to laser irradiation
- Experimental groups: Biofilms exposed to Er:YAG laser irradiation at defined fluence settings

Each experimental condition, defined by *Candida* species and laser setting, was performed in four independent replicates (*n* = 4). The same number of replicates (*n* = 4) was used for the corresponding control group for each species and incubation time point.

### 2.5. Evaluation of the Er:YAG Laser Effect on Growth Inhibition of Single-Species Candida Biofilms

Laser irradiation was performed using an Er:YAG laser system (Fotona LightWalker AT-S, Fotona, Slovenia) operating in pulsed mode at a fixed frequency of 2 Hz and pulse duration of 300 µs. The R16 handpiece was used in full-beam mode, providing a nominal circular spot diameter of 7 mm. Irradiation was performed in non-contact mode without air or water spray. The laser handpiece was mounted on a fixed stand and positioned perpendicular to the agar surface at a distance of 10 mm. A positioning template with 26 predefined irradiation points arranged in two concentric circles was placed beneath each Petri dish to standardize exposure locations. Following irradiation, plates were incubated at 37 °C and evaluated after 24, 48, 72, and 96 h. Growth inhibition zones (GIZs) were documented photographically and measured after 24, 48, 72, and 96 h using ImageJ-Fiji software (version 1.53j), calibrated against a metric reference. The laser settings, defined by fluence and corresponding average power at 2 Hz (pulse duration 300 µs), were as follows: fluence values of 0.3, 0.4, 0.5, 0.6, 0.7, 0.8, 0.9, 1.0, 1.5, 2.0, 2.5, 3.0, and 3.4 J/cm^2^, corresponding to average power outputs of 0.20, 0.30, 0.35, 0.45, 0.50, 0.60, 0.65, 0.75, 1.15, 1.50, 1.90, 2.30, and 2.60 W, respectively. Given a nominal beam diameter of 7 mm, the irradiated surface area was calculated as A = πr^2^, resulting in an area of 0.3848 cm^2^. Derived laser parameters, including power density, pulse energy, and total delivered energy, are summarized in Table 1 and Table 2. Fluence values were calculated based on the manufacturer-provided laser output parameters and nominal beam diameter. Independent verification of fluence at the sample surface using a power meter was not performed, and beam homogeneity was assumed based on the handpiece design specifications. Given a 7 mm beam diameter, the irradiated area was calculated as:

A = πr^2^ = π·(0.35 cm)^2^ = 0.384845 cm^2^.

The results of this are presented in Table 1. Laser parameters are noted in Table 2.

The Er:YAG laser system was operated in dual-pulse mode (manufacturer-defined MSP mode), delivering a sequence of two sub-pulses within each pulse envelope, as implemented in the Fotona LightWalker AT-S platform (Fotona d.o.o., Ljubljana, Slovenia). Irradiation was performed at a fixed repetition rate of 2 Hz. Consequently, during the 120 s exposure applied in the mature biofilm phase, each irradiated area received a total of 240 laser pulses. The R16 handpiece was used in full-beam mode, producing a nominal circular spot diameter of 7 mm. Independent experimental measurements of beam uniformity or spatial energy distribution were not performed. Beam homogeneity was therefore assumed based on the manufacturer’s optical design specifications for the full-beam handpiece, which are intended to provide a relatively uniform energy distribution across the irradiated area.

### 2.6. Evaluation of the Er:YAG Laser Effect on Elimination of Mature Single-Species Candida Biofilms

For the assessment of mature biofilm viability, *Candida* biofilms were allowed to develop for 96 h on Sabouraud dextrose agar at 37 °C. After incubation, plates were subjected to Er:YAG laser irradiation using the same system and general setup described above (2 Hz, 300 µs, non-contact mode, 10 mm distance, no air or water spray).

A custom template dividing the agar surface into four discrete, non-overlapping squares (12 × 12 mm each) was used to define irradiation areas. Each square was irradiated independently using one of the following fluence and power settings:

- Upper left: 0.8 J/cm^2^ (0.60 W)
- Upper right: 1.0 J/cm^2^ (0.75 W)
- Lower left: 1.5 J/cm^2^ (1.15 W)
- Lower right: 2.0 J/cm^2^ (1.50 W)

Each square was exposed for a fixed duration of 120 s. The distance between adjacent squares exceeded the nominal beam diameter, and the laser handpiece was repositioned between exposures to avoid overlap or unintended irradiation of neighboring areas. The template remained in place throughout the procedure to ensure consistent positioning and to minimize edge effects. No additional shielding was applied, but care was taken to prevent direct or reflected laser exposure outside the defined irradiation zones. Immediately after laser exposure, Rodac contact plates (IRR LAB-Agar, Sabouraud dextrose agar with chloramphenicol, 55 mm diameter) were applied to each treated area with gentle, standardized pressure for 10 s to obtain microbial imprints. The contact plates were then incubated for 24 h at 37 °C. After incubation, colonies were documented photographically under the same conditions used for the growth inhibition assessment, and viable counts were expressed as colony-forming units (CFUs) per imprint. The selection of fluence values for this phase was based on the growth inhibition results described in Section 3.1, which indicated measurable and stable inhibition at low to moderate fluence ranges. An exposure time of 120 s per square was chosen to standardize total energy delivery across all samples and to enhance detection of fluence-dependent differences in mature biofilm viability.

### 2.7. Statistical Analysis

Data were analyzed retrospectively, and all results are reported in the past tense. Normality of data distribution was assessed using the Shapiro–Wilk test, and homogeneity of variances was evaluated using Levene’s test. For each *Candida* species, one-way analysis of variance (ANOVA) was used to assess the effect of laser fluence on the diameter of growth inhibition zones (Phase 1) and on colony-forming unit (CFU) counts in mature biofilms (Phase 2). When ANOVA indicated a statistically significant overall effect (*p* < 0.05), post hoc multiple comparisons were performed. Tukey’s honestly significant difference (HSD) test was applied when assumptions of normality and homogeneity of variance were met. The Newman–Keuls test was used in cases where variance homogeneity was marginal but ANOVA assumptions were not violated sufficiently to preclude parametric testing. Outliers were evaluated using inspection of standardized residuals and boxplot analysis. Observations exceeding ±3 standard deviations from the group mean were flagged as potential outliers. No data points were excluded, as all values were traceable to valid experimental replicates and no technical or procedural errors were identified during data acquisition. Results are presented as mean ± standard deviation (SD). Statistical significance was defined as *p* < 0.05.

## 3. Results

### 3.1. Growth Inhibition of Candida Biofilms (Phase 1)

Quantitative assessment of growth inhibition was performed by measuring growth inhibition zone (GIZ) diameters at 24 h after Er:YAG irradiation. Mean GIZ values and standard deviations for all fluence levels and *Candida* species are summarized in Table 3. Subsequent observations at 48, 72, and 96 h were qualitative and were used to assess persistence of inhibition rather than changes in zone diameter.

For all tested species, Er:YAG irradiation produced measurable growth inhibition at all fluence levels tested (0.3–3.4 J/cm^2^). One-way ANOVA demonstrated a significant effect of fluence on GIZ diameter for each species (*p* < 0.05).

#### 3.1.1. *Candida albicans* ATCC 10231

*C. albicans* showed a clear fluence-dependent increase in GIZ diameter. At low fluence (0.3–0.5 J/cm^2^), mean GIZs ranged from approximately 5.5 to 5.6 mm at 24 h. At intermediate fluence (0.6–1.0 J/cm^2^), inhibition zones increased to approximately 6.5–6.8 mm. At higher fluence levels (1.5–3.4 J/cm^2^), mean GIZs ranged from approximately 6.9 to 7.4 mm. No visible regrowth into inhibited areas was observed up to 96 h.

#### 3.1.2. *Candida parapsilosis* ATCC 22019

*C. parapsilosis* exhibited pronounced growth inhibition with increasing fluence. Mean GIZ diameters increased from approximately 5.1 mm at 0.3 J/cm^2^ to approximately 7.7 mm at 3.4 J/cm^2^. The increase was most pronounced between 0.3 and 1.5 J/cm^2^, after which GIZ diameters showed a tendency toward plateauing. Inhibition zones remained clearly delineated throughout the 96 h observation period.

#### 3.1.3. *Candida krusei* ATCC 6258

*C. krusei* demonstrated smaller inhibition zones at low fluence levels. Mean GIZs ranged from approximately 5.0 to 5.3 mm between 0.3 and 0.6 J/cm^2^. A gradual increase was observed at higher fluence, reaching approximately 7.0 mm at 3.0–3.4 J/cm^2^. Inhibition zones persisted without visible reduction over time.

#### 3.1.4. *Candida glabrata* ATCC 90030

*C. glabrata* was the least responsive species in Phase 1. Mean GIZ diameters ranged from approximately 4.0 mm at 0.3 J/cm^2^ to approximately 5.2 mm at 3.4 J/cm^2^. Despite smaller inhibition zones, no regrowth was observed within inhibited areas during the 96 h observation period.

### 3.2. Reduction of Viable Cells in Mature Biofilms (Phase 2)

**Figure 1:**
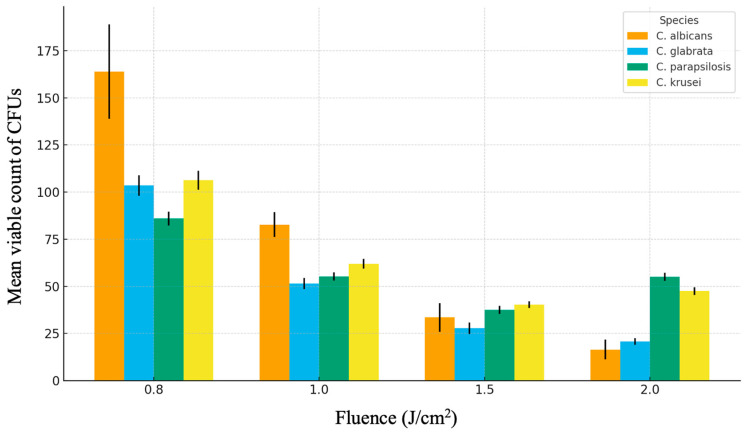
Mean viable counts of *Candida* colonies (CFUs) following Er:YAG laser irradiation at increasing fluence levels (0.8–2.0 J/cm^2^). Data represent mean ± SD for each species (*n* = 4). A progressive fluence-dependent reduction in viability is observed; most pronounced for *C. albicans* and *C. glabrata*.

Quantitative analysis of mature biofilm viability was performed using CFU counts obtained by contact imprinting after irradiation of 96 h biofilms. Mean CFU values with standard deviations are presented in Table 3, and graphical representation is shown in Figure 1. For all *Candida* species, Er:YAG irradiation resulted in statistically significant reductions in CFU counts compared with the lowest fluence tested (0.8 J/cm^2^) (one-way ANOVA, *p* < 0.05). The magnitude and pattern of reduction differed among species.

#### 3.2.1. *Candida albicans* ATCC 10231

Mean CFU counts decreased from 164.0 ± 25.1 at 0.8 J/cm^2^ to 82.8 ± 6.7 at 1.0 J/cm^2^, 33.5 ± 7.5 at 1.5 J/cm^2^, and 16.5 ± 5.2 at 2.0 J/cm^2^. This corresponds to an approximate 90 percent reduction relative to the lowest fluence.

#### 3.2.2. *Candida glabrata* ATCC 90030

For *C. glabrata*, CFU counts declined from 103.5 ± 5.4 at 0.8 J/cm^2^ to 51.5 ± 2.9 at 1.0 J/cm^2^, 27.8 ± 3.0 at 1.5 J/cm^2^, and 20.8 ± 1.7 at 2.0 J/cm^2^, representing an approximate 80 percent reduction.

#### 3.2.3. *Candida parapsilosis* ATCC 22019

*C. parapsilosis* showed a non-linear response. Mean CFU counts decreased from 86.0 ± 3.7 at 0.8 J/cm^2^ to 55.3 ± 2.2 at 1.0 J/cm^2^ and reached a minimum of 37.5 ± 2.1 at 1.5 J/cm^2^. At 2.0 J/cm^2^, CFU counts increased to 55.0 ± 2.2, indicating reduced efficacy at the highest fluence tested.

#### 3.2.4. *Candida krusei* ATCC 6258

A similar pattern was observed for *C. krusei*. CFU counts decreased from 106.3 ± 5.1 at 0.8 J/cm^2^ to 62.0 ± 2.6 at 1.0 J/cm^2^ and 40.3 ± 1.7 at 1.5 J/cm^2^, followed by an increase to 47.5 ± 2.1 at 2.0 J/cm^2^.

### 3.3. Summary of Results

Across both experimental phases, Er:YAG laser irradiation produced fluence-dependent inhibition of growth and reduction of viable cells in single-species *Candida* biofilms. The magnitude of the effect was species dependent. *C. albicans* and *C. glabrata* showed the most consistent reductions in mature biofilm viability, while *C. parapsilosis* and *C. krusei* demonstrated maximal effects at intermediate fluence levels, with reduced efficacy at the highest fluence tested.

#### Summary of Mature Biofilm Response

Altogether, Er:YAG irradiation at 0.8–2.0 J/cm^2^ reduced the viability of mature *Candida* biofilms, with the strongest disinfection in *C. albicans* and *C. glabrata. C. parapsilosis* and *C. krusei* were more resistant at the highest fluence, which points to the need for species adjusted laser parameters. This is summarized in Table 4 and Figure 1.

## 4. Discussion

The present in vitro study examined the effects of Er:YAG laser irradiation on the growth inhibition and viability reduction of single-species *Candida* biofilms using a large, homogeneously illuminated beam. The results demonstrate that Er:YAG irradiation can induce statistically significant, fluence-dependent reductions in biofilm growth and viable cell counts across multiple *Candida* species. However, given the modest sample size, simplified experimental model, and limited parameter space explored, these findings should be interpreted as evidence of controlled antifungal disinfection rather than definitive proof of eradication or clinical efficacy.

### 4.1. Interpretation of the Primary Findings

Both experimental phases showed a consistent association between increasing fluence and antifungal effect, as reflected by larger growth inhibition zones in early biofilms and lower CFU counts in mature biofilms. These outcomes indicate that Er:YAG irradiation interferes with *Candida* biofilm development and persistence under defined laboratory conditions. Nevertheless, the statistical analyses applied in this study primarily demonstrate differences between fluence levels and controls, without detailed exploration of effect size distributions or mechanistic correlates. Consequently, conclusions regarding biological relevance should remain proportionate to the scope of the data.

The persistence of growth inhibition over 96 h suggests that the initial laser exposure produced durable alterations in biofilm structure or viability. However, because inhibition zone diameters were quantitatively assessed only at 24 h, later time points served to confirm stability rather than to provide additional quantitative resolution. While this approach is common in in vitro biofilm studies, it limits temporal insight into dynamic regrowth processes [10,17].

### 4.2. Species-Specific Susceptibility Patterns

Clear interspecies differences were observed in both growth inhibition and mature biofilm viability reduction. *Candida albicans* and *C. parapsilosis* showed the greatest susceptibility to Er:YAG irradiation, whereas *C. glabrata* exhibited consistently lower responses, and *C. krusei* demonstrated an intermediate pattern. These findings align with prior reports indicating that Candida species differ substantially in biofilm architecture, extracellular matrix composition, and intrinsic stress tolerance [2,4,10,12]. Several biological factors may contribute to these differences. For example, *C. glabrata* is known to form denser, more compact biofilms with lower metabolic activity, which may reduce susceptibility to physical or thermal stress [2,4]. In contrast, *C. parapsilosis* often forms thinner biofilms with higher surface exposure, potentially facilitating energy absorption [10,17]. Differences in extracellular matrix hydration, polysaccharide composition, and expression of heat shock or stress response proteins may also influence laser responsiveness [4,15,18]. Importantly, these explanations remain hypothetical within the context of the present study, as no direct structural, biochemical, or molecular analyses were performed. The speculative nature of these interpretations should therefore be emphasized.

Most previously published in vitro studies investigating the antifungal effects of Er:YAG irradiation on *Candida* biofilms have employed narrow fiber tips or small spot diameters, typically resulting in highly localized energy delivery and steep energy gradients [10,11,12,13,19]. Under such conditions, antifungal effects are often limited to focal ablation or superficial disruption, and reported outcomes frequently depend on higher fluence values or short exposure times. In contrast, the present study specifically explored low-fluence irradiation delivered over a comparatively large, homogeneously illuminated area using a 7 mm full-beam handpiece. This approach minimizes focal overheating and emphasizes distributed photothermal and micro-expansive effects rather than localized ablation. The observed fluence-dependent inhibition and partial biofilm disinfection at relatively low energy densities therefore extend existing literature by demonstrating that broad-beam Er:YAG irradiation can achieve measurable antifungal effects under conditions that differ substantially from conventional narrow-fiber protocols.

### 4.3. Effects on Mature Biofilms and Dose–Response Considerations

Mature 96 h biofilms represent a more resistant and clinically relevant phenotype than early biofilms, due to increased matrix complexity, reduced metabolic activity, and the presence of persister cells [3,4]. In this context, the observed reductions in CFU counts following Er:YAG irradiation indicate that laser energy can penetrate at least superficial biofilm layers and reduce viability. However, the absence of complete eradication across all tested conditions highlights the inherent limitations of single-modality physical disinfection in mature biofilms. The non-linear responses observed for *C. parapsilosis* and *C. krusei*, with partial CFU rebound at the highest fluence tested, underscore the complexity of dose–response relationships in biofilm systems. Increasing fluence does not necessarily translate into proportionally greater antimicrobial effects. Possible contributing factors include localized vapor formation that limits deeper energy penetration, heterogeneous biofilm density, or stress-induced survival mechanisms activated at higher energy exposures [17,18,19]. Without direct measurement of temperature gradients, matrix disruption, or cell viability distribution within the biofilm, these explanations remain tentative.

Absorption of Er:YAG laser radiation at 2940 nm is strongly dependent on water content, which is a critical determinant of energy deposition within microbial biofilms. In the present agar-based model, biofilm hydration is influenced by both the intrinsic water content of the extracellular matrix and the moisture retained within the agar substrate. Variations in surface hydration or partial drying of the biofilm during incubation may therefore alter local absorption characteristics and energy transfer. Increased hydration may enhance laser absorption and promote micro-expansive effects within the biofilm matrix, whereas partial dehydration could reduce effective energy coupling and limit antifungal efficacy. Because hydration state was not actively controlled or quantified in this study, its contribution to the observed species- and fluence-dependent effects cannot be excluded and should be considered when interpreting the results. Future studies incorporating controlled hydration conditions or real-time moisture monitoring may help clarify the role of water content in Er:YAG-mediated biofilm disruption.

### 4.4. Disinfection Versus Eradication

A critical conceptual distinction highlighted by the present findings is that between disinfection and eradication. Although statistically significant reductions in CFU counts were achieved, no laser parameter resulted in complete elimination of *Candida* biofilms. This outcome is consistent with previous Er:YAG and laser-based disinfection studies, which generally report partial reductions rather than sterilization, particularly in structured biofilms [10,15,16,20,21,22,23,24,25,26,27,28]. Recognizing this distinction is important to avoid unrealistic expectations regarding laser-based antifungal strategies. In the context of biofilm-associated infections, reduction of microbial burden may still be clinically meaningful, particularly when combined with mechanical cleaning or pharmacological agents. However, the present study does not provide evidence to support Er:YAG irradiation as a standalone eradication method.

### 4.5. Translational Implications and Caution

While the antifungal effects observed in this study suggest potential relevance for oral surfaces colonized by *Candida*, the experimental model remains far removed from clinical reality. The study did not assess temperature rise, cytotoxicity to host cells, or effects on dental materials or mucosal tissues. Consequently, extrapolation to clinical protocols for denture stomatitis, peri-implantitis, or mucosal candidiasis should be approached with caution.

Previous studies have suggested that Er:YAG irradiation may be safely applied to oral tissues at low fluence levels [6,9,15], and that combination approaches with antiseptics or irrigants may enhance antimicrobial outcomes [17,18,19]. However, such considerations remain speculative in the absence of direct safety and efficacy data from the present work.

Although the present study employed single-species *Candida* biofilms, the oral cavity is characterized by complex polymicrobial communities that include streptococci, anaerobes, *Staphylococcus aureus*, and other bacterial taxa that interact structurally and metabolically with *Candida* spp. [29]. Such interactions can influence biofilm architecture, matrix composition, and susceptibility to physical disruption. The partial reductions observed in the current single-species model may therefore underestimate or overestimate effects occurring in polymicrobial biofilms, where cooperative or competitive interactions could modify laser responsiveness. Acknowledging this complexity further underscores the need for future studies using multispecies biofilm models that more closely approximate clinical conditions.

### 4.6. Limitations

Several limitations of this study should be acknowledged. First, only reference ATCC strains were used, and no clinical isolates were included. While this enhances standardization and reproducibility, it limits insight into strain-level variability encountered in clinical infections. Second, the use of single-species biofilms grown on agar does not replicate the multispecies composition, salivary conditioning, or mechanical forces present in the oral cavity [1,2]. Third, the laser parameter space was restricted to a single pulse frequency (2 Hz) and pulse duration (300 µs), and the number of replicates per condition was modest (*n* = 4). Fourth, laser output parameters were based on manufacturer specifications, without independent verification of fluence at the sample surface or quantitative assessment of beam homogeneity. Finally, no cytotoxicity, thermal, or material compatibility assessments were performed, precluding conclusions about clinical safety.

### 4.7. Future Directions

Future investigations should expand upon these findings by incorporating clinical *Candida* isolates, multispecies biofilm models, and substrates that better reflect oral materials, such as acrylic resin or titanium. Detailed thermal monitoring and host cell viability assays will be essential to define safe operating windows. Repeated or cumulative irradiation protocols, as well as combination strategies with antifungal agents or antiseptics, may help overcome the partial reductions observed in mature biofilms. Ultimately, controlled in vivo studies will be required to determine whether the in vitro disinfection effects demonstrated here translate into meaningful clinical benefits.

## 5. Conclusions

Within the limitations of this in vitro study, Er:YAG laser irradiation delivered in non-contact mode with a 7 mm full-beam handpiece produced fluence-dependent inhibition of growth and measurable reductions in the viability of single-species *Candida* biofilms grown on agar. Stable growth inhibition zones and significant decreases in colony-forming unit counts were observed for all tested species, with the magnitude of the effect varying among *C. albicans*, *C. glabrata*, *C. parapsilosis*, and *C. krusei*. The results indicate that low to moderate fluence Er:YAG irradiation can achieve partial disinfection of both early and mature *Candida* biofilms under controlled laboratory conditions. Species-specific differences in response were observed, and for *C. parapsilosis* and *C. krusei* the data suggest that increasing fluence beyond certain levels did not further enhance biofilm reduction; however, this observation is based on a limited number of fluence settings and should be interpreted cautiously. Overall, the findings support the conclusion that Er:YAG irradiation can reduce fungal biofilm burden in vitro, but they do not demonstrate eradication or provide evidence for clinical efficacy or safety. Further studies using multispecies biofilm models, clinical isolates, host-relevant substrates, and in vivo or ex vivo systems are required before implications for clinical management of oral *Candida*-associated conditions can be drawn.

## Figures and Tables

**Table 1:** Parameters of *Candida* biofilm growth inhibition (7 mm spot size; 2 Hz).

Fluence [J/cm^2^]	Power [W]	Power Density [W/cm^2^]	Pulse Energy [J]	Total E (W × t) [J]
0.30	0.20	0.52	0.12	24.0
0.40	0.30	0.78	0.15	36.0
0.50	0.35	0.91	0.19	42.0
0.60	0.45	1.17	0.23	54.0
0.70	0.50	1.30	0.27	60.0
0.80	0.60	1.56	0.31	72.0
0.90	0.65	1.69	0.35	78.0
1.00	0.75	1.95	0.38	90.0
1.50	1.15	2.99	0.58	138.0
2.00	1.50	3.90	0.77	180.0
2.50	1.90	4.94	0.96	228.0
3.00	2.30	5.98	1.15	276.0
3.40	2.60	6.76	1.31	312.0

**Table 2:** Elimination of mature *Candida* biofilms—fluence and power density parameters (7 mm spot size; 2 Hz; 120 s).

Fluence [J/cm^2^]	Power [W]	Power Density [W/cm^2^]	Pulse Energy [J]	Total E (W × t) [J]
0.80	0.60	1.56	0.31	72.0
1.00	0.75	1.95	0.38	90.0
1.50	1.15	2.99	0.58	138.0
2.00	1.50	3.90	0.77	180.0

**Table 3:** Growth inhibition zone (GIZ) diameters (mm) of single-species *Candida* biofilms 24 h after Er:YAG laser irradiation at increasing fluence levels. Values are expressed as mean ± SD.

Fluence (J/cm^2^)	*C. albicans*	*C. glabrata*	*C. parapsilosis*	*C. krusei*
0.3	5.5 ± 0.2	4.0 ± 0.1	5.1 ± 0.2	5.0 ± 0.2
0.4	5.5 ± 0.2	4.2 ± 0.1	5.3 ± 0.2	5.1 ± 0.2
0.5	5.6 ± 0.2	4.4 ± 0.1	5.5 ± 0.2	5.2 ± 0.2
0.6	6.5 ± 0.3	4.6 ± 0.1	6.1 ± 0.3	5.3 ± 0.2
0.7	6.6 ± 0.3	4.7 ± 0.1	6.4 ± 0.3	5.6 ± 0.2
0.8	6.7 ± 0.3	4.8 ± 0.1	6.7 ± 0.3	5.9 ± 0.2
0.9	6.8 ± 0.3	4.9 ± 0.1	6.9 ± 0.3	6.1 ± 0.2
1.0	6.8 ± 0.3	5.0 ± 0.1	7.0 ± 0.3	6.2 ± 0.2
1.5	6.9 ± 0.3	5.1 ± 0.1	7.3 ± 0.3	6.5 ± 0.2
2.0	7.1 ± 0.3	5.1 ± 0.1	7.4 ± 0.3	6.7 ± 0.2
2.5	7.2 ± 0.3	5.2 ± 0.1	7.6 ± 0.3	6.9 ± 0.2
3.0	7.3 ± 0.3	5.2 ± 0.1	7.7 ± 0.3	7.0 ± 0.2
3.4	7.4 ± 0.3	5.2 ± 0.1	7.7 ± 0.3	7.0 ± 0.2

**Table 4:** Reduction in *Candida* viability with increasing fluence (J/cm^2^). Mean ± SD values are shown for each species (*n* = 4).

Species	Concentration	Mean	SD
*C. albicans*	0.8	164	25.07322609
	1	82.75	6.652067348
	1.5	33.5	7.549834435
	2	16.5	5.196152423
*C. glabrata*	0.8	103.5	5.446711546
	1	51.5	2.886751346
	1.5	27.75	2.986078811
	2	20.75	1.707825128
*C. parapsilosis*	0.8	86	3.651483717
	1	55.25	2.217355783
	1.5	37.5	2.081665999
	2	55	2.160246899
*C. krusei*	0.8	106.25	5.057996968
	1	62	2.581988897
	1.5	40.25	1.707825128
	2	47.5	2.081665999

## Data Availability

All generated data is available in the manuscript.
